# LONGITUDINAL STUDIES OF COGNITIVE AGING: STUDY DESIGNS TO OPTIMIZE STATISTICAL POWER TO DETECT COGNITIVE DECLINE

**DOI:** 10.1093/geroni/igae098.4019

**Published:** 2024-12-31

**Authors:** Jasmin Duehring, Lawrence Ong, Steven Edland

**Affiliations:** University of California San Diego, La Jolla, California, United States; University of Callifornia San Diego, La Jolla, California, United States; University of California San Diego, La Jolla, California, United States

## Abstract

Practice effects in longitudinal studies of cognitive function in aging and Alzheimer’s disease refer to the observation that performance on cognitive measures may improve on repeated assessments, because of increased familiarity with testing procedures and content of the tests. We have previously shown that a clinical trial run-in period intended to wash out practice effects can dramatically improve the efficiency of clinical trials. We now show that a run-in period can dramatically improve the efficiency and statistical power of longitudinal cohort studies of cognitive aging as well. Data are 1,094 participants with a baseline diagnosis of amnestic mild cognitive impairment (aMCI) enrolled in the National Alzheimer Coordinating Center (NACC) cohort (mean age 74.8, range 60 – 97, 44.1% female). In these data, the signal-to-noise in annual change after a run-period was approximately twice (2.4-fold) that observed without a run-in period. This increased signal translates to an 80% reduction in sample size required to detect a given effect of exposure, or, conversely, to an increase in statistical power given available sample size. For example, in the NACC cohort, p-values for detection of a one year change were p=0.086 without run-in, and p=8e-5 with a run-in period. Incorporating an early run-in period to longitudinal cohort studies of aging and Alzheimer’s disease can dramatically improve the statistical power of these studies to detect predictors of cognitive decline.

